# Metastatic MTLn3 and non-metastatic MTC adenocarcinoma cells can be differentiated by *Pseudomonas aeruginosa*

**DOI:** 10.1242/bio.20133632

**Published:** 2013-07-03

**Authors:** Matthew J. Novotny, Dacie R. Bridge, Karen H. Martin, Scott A. Weed, Robert B. Wysolmerski, Joan C. Olson

**Affiliations:** 1 Department of Microbiology, Immunology and Cell Biology, West Virginia University Health Sciences Center, Morgantown, WV 26506-9177, USA; 2 Department of Neurobiology and Anatomy, West Virginia University Health Sciences Center, Morgantown, WV 26506-9128, USA; 3 Mary Babb Randolph Cancer Center, West Virginia University Health Sciences Center, Morgantown, WV 26506-9300, USA; ‡ Present address: Department of Microbiology and Immunology, Uniformed Services University of the Health Sciences, Bethesda, MD 20814, USA

**Keywords:** Tumor metastasis, MTC and MTLn3 cells, *Pseudomonas aeruginosa*, Rho GTPase, Cell migration

## Abstract

Cancer patients are known to be highly susceptible to *Pseudomonas aeruginosa* (*Pa*) infection, but it remains unknown whether alterations at the tumor cell level can contribute to infection. This study explored how cellular changes associated with tumor metastasis influence *Pa* infection using highly metastatic MTLn3 cells and non-metastatic MTC cells as cell culture models. MTLn3 cells were found to be more sensitive to *Pa* infection than MTC cells based on increased translocation of the type III secretion effector, ExoS, into MTLn3 cells. Subsequent studies found that higher levels of ExoS translocation into MTLn3 cells related to *Pa* entry and secretion of ExoS within MTLn3 cells, rather than conventional ExoS translocation by external *Pa*. ExoS includes both Rho GTPase activating protein (GAP) and ADP-ribosyltransferase (ADPRT) enzyme activities, and differences in MTLn3 and MTC cell responsiveness to ExoS were found to relate to the targeting of ExoS-GAP activity to Rho GTPases. MTLn3 cell migration is mediated by RhoA activation at the leading edge, and inhibition of RhoA activity decreased ExoS translocation into MTLn3 cells to levels similar to those of MTC cells. The ability of *Pa* to be internalized and transfer ExoS more efficiently in association with Rho activation during tumor metastasis confirms that alterations in cell migration that occur in conjunction with tumor metastasis contribute to *Pa* infection in cancer patients. This study also raises the possibility that *Pa* might serve as a biological tool for dissecting or detecting cellular alterations associated with tumor metastasis.

## Introduction

The relationship between bacterial infections and cancer is complex. Bacteria, such as *Helicobacter pylori*, are a proven carcinogen in the development of gastric cancer and mucosa-associated lymphoid tissue lymphoma (Crowe, 2005; Montalban et al., 2001). Other bacteria, such as *Salmonella typhi*, *Streptococcus bovis* and *Chlamydia pneumoniae* have been linked to the induction of specific types of cancers in association with chronic infection, inflammation or immune suppressive mechanisms (Dutta et al., 2000; Gold et al., 2004; Koyi et al., 2001; Kuper et al., 2000). Bacteria and their toxins have also been used to treat cancer. Coley's vaccine, comprised of live or heat-killed *Streptococcus pyogenes* and *Serratia marcescens*, was historically used to limit many forms of cancers, presumably by engaging an immune response that protected against tumors (McCarthy, 2006). More recently, modified bacteria, bacterial toxins and re-engineered immunotoxins that target tumors or tumor-specific antigens have been used clinically to preferentially kill tumor-associated cells (Patyar et al., 2010). In addition to the role of bacteria in tumor induction or treatment, physical and immunological compromises associated with cancer or cancer treatments make cancer patients highly susceptible to bacterial infection (Benharroch and Osyntsov, 2012). Relationships between bacterial infection and tumor induction introduce the notion that there might be commonalities in mechanisms of bacterial pathogenesis and tumor induction that can be applied to an understanding of neoplastic processes.

*Pseudomonas aeruginosa* (*Pa*) is a Gram-negative, predominantly extracellular, opportunistic pathogen that frequently infects cancer patients. *Pa* maintains a broad range of virulence factors that contribute to its pathogenicity, but among these, the type III secretion (T3S) system is recognized to be integral to the initiation of *Pa* infection and is associated with poor clinical outcomes (Hauser, 2009). The T3S system is a needle-like nanostructure made by many Gram-negative bacteria that allows the direct translocation of proteins or ‘effectors’ from the bacterial cytosol to the host cell surface (Cornelis, 2010). T3S effectors are then internalized into host cells through a bacterially formed ‘translocon’ channel in eukaryotic cell membranes. Within the cell, T3S effectors manipulate host cell function in a bacterial specific manner to facilitate bacterial growth and survival. The importance of T3S in the establishment of *Pa* infection is supported by the findings that immunity induced against the T3S translocon protein, PcrV, protects against *Pa* infection, and that cellular susceptibility to *Pa* infection parallels cellular sensitivity to T3S (Bridge et al., 2010; McGuffie et al., 1999; Rucks and Olson, 2005; Sawa et al., 1999).

*Pa* utilizes T3S to disrupt normal host cell function and promote infection through four identified effectors, ExoS, ExoT, ExoU and ExoY. ExoS and ExoT are homologous, bifunctional proteins that include Rho GTPase activating protein (GAP) and ADP-ribosyltransferase (ADPRT) activities (Goehring et al., 1999; Iglewski et al., 1978; Krall et al., 2000; Yahr et al., 1996). The GAP activity of ExoS and ExoT functions in a similar manner to inhibit host cell Rho family GTPase activity and alter actin dynamics to prevent *Pa* internalization (Garrity-Ryan et al., 2000). ExoS plays a more pronounced role in *Pa* pathogenesis than ExoT (Shaver and Hauser, 2004), and this coincides with ExoS-ADPRT activity having specificity for multiple cellular proteins, including certain Ras family proteins (McGuffie et al., 1998; Fraylick et al., 2002b; Henriksson et al., 2002), ERM (ezrin, moesin and radixin) proteins (Maresso et al., 2004; McGuffie et al., 1998), vimentin (Coburn et al., 1989), and cyclophilin A (DiNovo et al., 2006). The substrate specificity of ExoT-ADPRT activity in comparison is limited to Crk proteins (Sun and Barbieri, 2003). ExoU has phospholipase A_2_ activity that causes cell lysis and is associated with the most virulent *Pa* infections (Sato et al., 2003; Shaver and Hauser, 2004). ExoY has adenylate cyclase activity and appears to play a limited role in *Pa* pathogenesis (Vance et al., 2005; Yahr et al., 1998). Translocation of T3S effectors across host cell membranes is the least understood stage in T3S but is known to require three proteins in *Pa*: membrane channel forming PopB and PopD and channel assembling PcrV (Mueller et al., 2008).

Examination of eukaryotic cell properties that influence T3S translocation and responsiveness to ExoS found that most cell lines were sensitive to ExoS toxicity, but different degrees of toxicity were observed (McGuffie et al., 1999; Rucks et al., 2003). Two cell lines, polarized confluent epithelial cells and undifferentiated HL-60 promyelocytic cells, have been identified to be resistant to *Pa*-T3S and ExoS toxicity (McGuffie et al., 1999; Rucks et al., 2003; Rucks and Olson, 2005). Notably, tumor-derived cell lines are generally highly sensitive to ExoS toxicity (McGuffie et al., 1999). More recent studies of human HT-29 colon and T24 bladder carcinoma cell lines identified a relationship between the leading edge of migrating epithelial cells and sensitivity to *Pa*-T3S and infection (Bridge et al., 2012; Bridge et al., 2010). The finding that carcinoma derived cell lines are highly sensitive to *Pa*-T3S and ExoS toxicity, while healthy polarized epithelial monolayers are resistant to both, introduces the possibility that responsiveness to ExoS toxicity might provide insight into mechanistic properties linked to carcinogenesis.

The purpose of this study was to characterize properties of carcinogenic cells that contribute to their increased sensitivity to T3S translocated ExoS and the establishment of *Pa* infection. Realizing the complexity of tumor development, this study focused on two closely related tumor cell lines, MTC and MTLn3 cells. These cell lines were derived from subcutaneous implantation of the rat mammary 13762 adenocarcinoma cell line into Fisher 344 rats (Neri et al., 1982), but differ in that MTC cells are non-metastatic and MTLn3 cells acquired high metastatic potential. Based on the premise that cell migration influences sensitivity to *Pa* infection, we examined whether alterations in cell migration associated with tumor metastasis might be a factor in influencing susceptibility to *Pa* infection.

Our studies found that ExoS was translocated more efficiently into highly metastatic MTLn3 cells as compared to non-metastatic MTC cells, consistent with tumor metastasis enhancing sensitivity to *Pa* infection. When the mechanism underlying differences in ExoS translocation between the two cell lines was examined, increased ExoS translocation into MTLn3 cells occurred in conjunction with increased *Pa* entry and secretion of ExoS within MTLn3 cells. Rho activation is increased at the leading edge of MTLn3 cells (El-Sibai et al., 2007; El-Sibai et al., 2008), and inhibition of Rho activity in MTLn3 cells decreased ExoS translocation to levels similar to that of MTC cells. These are the first studies to demonstrate that Rho activation in metastatic MTLn3 cells alters sensitivity to *Pa* infection and mechanistically link alterations in cell migration associated with tumor metastasis to increased sensitivity to *Pa* infection.

## Results

### Highly metastatic MTLn3 cells allow greater *Pa*-T3S translocation than non-metastatic MTC cells

**Fig. 1.**
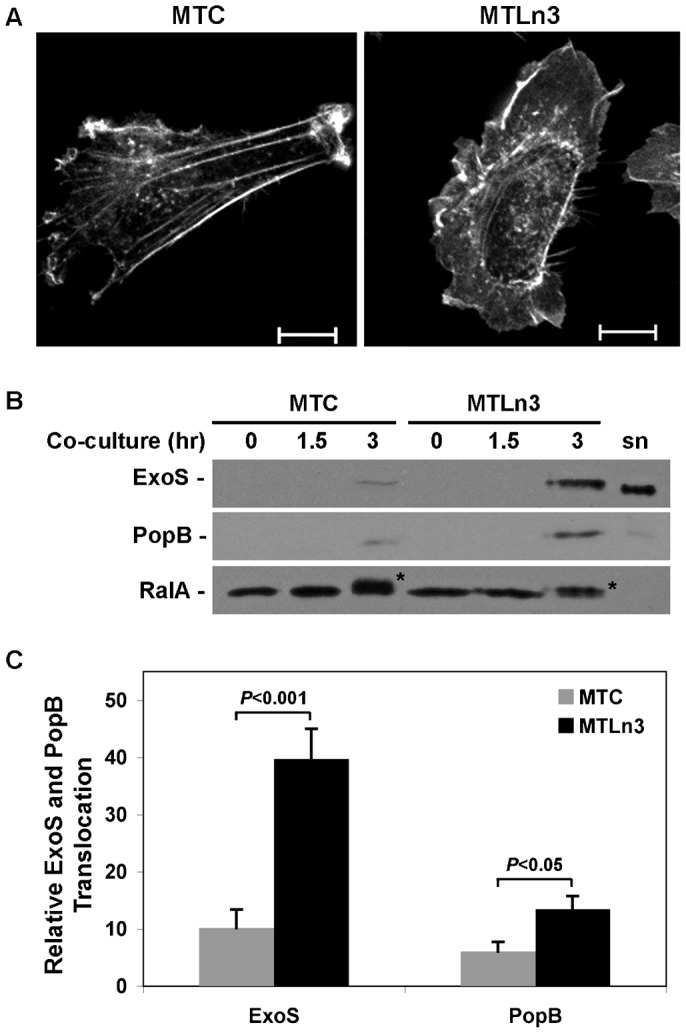
Highly metastatic MTLn3 cells exhibit a different migratory phenotype and allow greater *Pa*-T3S translocation than non-metastatic MTC cells. (**A**) MTC and MTLn3 cell morphology was compared under steady state conditions (5% FBS) in the absence of *Pa*. Cells were stained with Phalloidin-TRITC to detect actin (white). Scale bar: 10 µm. (**B**) MTC and MTLn3 cells were co-cultured with strain PA103ΔUT expressing plasmid encoded HA tagged wild type ExoS (*Pa* ExoS-WT) for the indicated times. Cells were harvested, fractionated and T3S translocation of ExoS and PopB into membrane fractions was assayed by immunoblot analysis based on equal protein loading. RalA modification served as a functional read-out of translocated ExoS-ADPRT activity, and asterisks mark ADP-ribosylated RalA. Total RalA served as a membrane fraction loading control. (sn) T3S induced *Pa* ExoS-WT culture supernatant was used as a molecular marker for ExoS and PopB. (**C**) T3S translocated ExoS and PopB were quantified by densitometry, and significant differences in ExoS and PopB translocation between MTC and MTLn3 cells are indicated. Results represent the mean ± s.e.m. of five independent experiments.

To examine if tumor metastasis influenced sensitivity to *Pa* infection, we compared the efficiency of *Pa*-T3S translocation into non-metastatic MTC cells with that of highly metastatic MTLn3 cells. Detection of *Pa*-T3S translocation into eukaryotic cells requires a 1.5 to 3.5 hr co-culture period, depending on the cell line and the method of analysis (Bridge et al., 2012; Bridge et al., 2010). To adapt *Pa* co-culture conditions to studies of MTC and MTLn3 cells, indicated infections were performed in medium containing 5% FBS (steady state conditions) to allow cells to move randomly during the co-culture period. Examination of MTC and MTLn3 cell morphology under steady state conditions found MTC cells to have an elongated polarized morphology and a well-developed leading edge, typical of mesenchymal migration (Fig. 1A). MTLn3 cells in comparison were more rounded, lacked head-to-tail polarity, and had a broad thin lamellar region demarcated by actin. These morphological findings are similar to those previously observed for MTC or MTLn3 cells following starvation and stimulation with 5% serum (Shestakova et al., 1999), and document that metastatic properties of MTC and MTLn3 cells correspond with different migratory phenotypes.

To compare *Pa*-T3S translocation in MTC and MTLn3 cells, sub-confluent monolayers were co-cultured with strain PA103ΔUT expressing plasmid encoded HA-tagged wild type ExoS (*Pa* ExoS-WT) for increasing times (0, 1.5 or 3 hr). T3S translocation was assayed by monitoring ExoS effector transfer into MTC or MTLn3 cell membrane fractions based on immunoblot analysis, which was then quantified by densitometry. As shown in Fig. 1B, no ExoS translocation was detected in uninfected (0) cells or in either cell line after co-culture with *Pa* ExoS-WT for 1.5 hr. Following a 3 hr co-culture period, ExoS translocation was detected in both cell lines, but significantly (*P*<0.001) higher levels of ExoS translocation (4-fold) were detected in MTLn3 cells as compared to MTC cells (Fig. 1C). ExoS immunoblots were also probed for RalA, which served as a membrane fraction loading control and is a substrate of ExoS-ADPRT activity. RalA can be ADP-ribosylated by *Pa* ExoS-WT at two to three sites, with Arg52 functioning as a preferred site of ExoS ADP-ribosylation, and Arg135 and Arg161 functioning as secondary sites of ADP-ribosylation (Fraylick et al., 2002a). ADP-ribosylation by ExoS causes RalA to shift in mobility in a manner dependent on the number of sites of ADP-ribosylation (Bridge et al., 2012; Fraylick et al., 2002a), and the different shift in RalA mobility detected between MTC and MTLn3 cells (Fig. 1B, noted by asterisks) can be explained by RalA being ADP-ribosylated at more sites in MTC cells.

To assess if MTLn3 cells had enhanced *Pa*-T3S translocation in general, duplicate immunoblots were probed for the T3S translocon protein, PopB. Similar to ExoS, significantly (*P*<0.05) more PopB was detected in the membrane fraction of MTLn3 as compared to MTC cells after a 3 hr co-culture period (Fig. 1B,C). Both cell lines remained >90% viable following co-culture with *Pa* ExoS-WT, indicating that differences in T3S translocation did not relate to ExoS cytotoxicity. T3S translocation is dependent on *Pa* contact with eukaryotic cells, but differences in T3S translocation did not relate to more efficient *Pa* binding to MTLn3 cells, as an approximately equal number of *Pa* (3.5±1.3 and 3.6±1.2) bound per MTLn3 and MTC cell, respectively. Collectively these studies found that T3S translocation was more efficient in metastatic MTLn3 cells than non-metastatic MTC cells, and provided evidence that ExoS ADP-ribosylation of RalA differed between the two cell lines.

### Use of ExoS enzyme activity to characterize mechanisms underlying differences in T3S translocation in MTC and MTLn3 cells

ExoS is a bi-functional T3S effector having N-terminal GAP activity, defined by arginine residue 146 (R146), and C-terminal ADPRT activity, defined by glutamic acid residues 379 and 381 (E379 and E381) (reviewed by Barbieri and Sun, 2004). Both activities can alter ExoS translocation into eukaryotic cells (Aili et al., 2008; Bridge et al., 2012; Cisz et al., 2008; Fraylick et al., 2001). In turn, inactivation of ExoS-GAP or ADPRT activity has proven useful in dissecting cellular mechanisms that underlie T3S translocation and *Pa* infection (Bridge et al., 2012). To determine if mutations that inactivate ExoS-GAP or ADPRT activities might provide insight into mechanisms underlying increased ExoS translocation into metastatic MTLn3 cells, MTC and MTLn3 cells were co-cultured for 3 hr with strain PA103ΔUT expressing plasmid encoded ExoS-HA with an R146A mutation, ExoS-GAP(−), or E379A and E381A mutations, ExoS-ADPRT(−). T3S translocation was assayed based on ExoS and PopB transfer into membrane fractions by immunoblot analysis, and this was compared with translocation of *Pa* ExoS-WT, a *Pa* pUCP vector control, and uninfected cells.

**Fig. 2.**
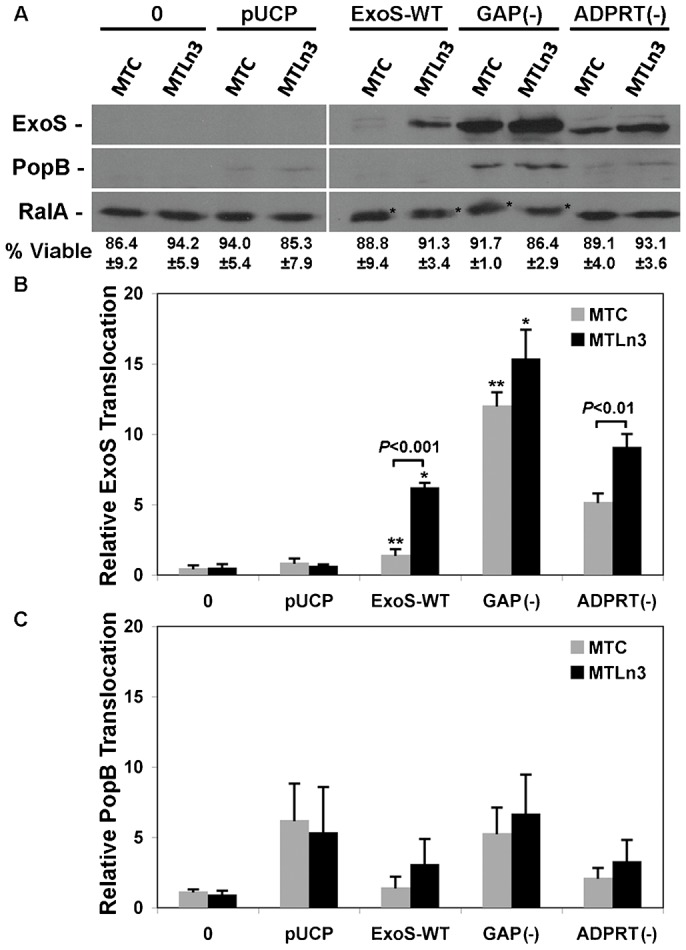
ExoS-GAP activity determines differences in T3S translocation in MTC and MTLn3 cells. (**A**) MTC and MTLn3 cells were co-cultured for 3 hr with *Pa* ExoS-WT, *Pa* expressing ExoS with a R146A GAP(−) mutant, or *Pa* expressing ExoS with an E379A and E381A ADPRT(−) mutant. Controls include uninfected cells (0) or cells co-cultured with a *Pa* pUCP plasmid control. Cells were harvested, fractionated and T3S translocation of ExoS and PopB into membrane fractions was assayed as in Fig. 1. (**B**) T3S translocated ExoS following co-culture with the indicated *Pa* strain was quantified by densitometry, and the mean ± s.e.m. of three independent experiments are represented. Significant differences between MTC and MTLn3 translocation of ExoS-WT and ExoS-ADPRT(−) are indicated. No significant difference in ExoS-GAP(−) translocation between MTC and MTLn3 cells was observed. Asterisks mark significant differences in ExoS-WT and ExoS-GAP(−) translocation into MTC cells (**P<0.001), or in ExoS-WT and ExoS-GAP(−) translocation into MTLn3 cells (*P<0.01). A significant (*P*<0.01) increase in translocation of ExoS-ADPRT(−) as compared to ExoS-WT was also detected in MTC cells, which is not indicated in Fig. 2B for simplicity. (**C**) T3S translocated PopB following co-culture with the indicated *Pa* strain was quantified by densitometry, and the mean ± s.e.m. of three independent experiments are represented. Differences observed in PopB translocation did not gain statistical significance.

As represented in Fig. 2A and quantified in 2B, inactivation of ExoS-GAP activity significantly enhanced ExoS translocation into both cell lines, as compared to *Pa* ExoS-WT, and allowed ExoS translocation into MTC cells to closely approximate that of MTLn3 cells. Inactivation of ExoS-ADPRT activity also caused a modest, but significant (*P*<0.01) increase in ExoS translocation into MTC cells when compared to *Pa* ExoS-WT (not noted in Fig. 2B). The increase in ExoS translocation into MTLn3 cells caused by inactivation of ExoS-ADPRT activity did not gain statistical significance when compared to *Pa* ExoS-WT. Comparisons between MTC and MTLn3 cells confirmed a significant increase in translocation of ExoS-WT (*P*<0.001) and ExoS-ADPRT(−) (*P*<0.01) into MTLn3 as compared to translocation of the respective effectors in MTC cells. No significant difference in ExoS-GAP(−) translocation was detected between the two cells lines. Alterations in PopB translocation caused by inactivation of ExoS-GAP or ADPRT activity generally paralleled alterations in ExoS translocation but were less pronounced and did not gain statistical significance (Fig. 2C). The *Pa* pUCP vector control included in this study maintains a functional T3S system but lacks T3S effectors. Consistent with these properties, PopB but not ExoS translocation was detected following co-culture with *Pa* pUCP. As in Fig. 1, alterations in ExoS translocation did not relate to cytotoxic effects of *Pa* strains, and differences in the efficiency of ExoS ADP-ribosylation of RalA were still evident between the two cell lines (Fig. 2A).

The most important findings of these analyses were that: 1) ExoS-GAP activity, and to lesser extent ExoS-ADPRT activity interfered with ExoS translocation into MTC and MTLn3 cells, indicating that ExoS was able to modulate T3S translocation by two independent mechanisms. 2) Inactivation of ExoS-GAP activity nullified differences in ExoS translocation between MTC and MTLn3 cells, indicating that differences in ExoS translocation between the two cells lines related to their reactivity to ExoS-GAP activity. This led to our examining whether ExoS-GAP activity might serve as a tool to further characterize cellular differences that contribute to metastatic properties of MTLn3 cells.

### ExoS-GAP activity differentially alters MTC and MTLn3 cell morphology

Effects of ExoS-GAP activity on MTC and MTLn3 cell function were further examined by immunofluorescence microscopy (IF), following co-culture with *Pa* expressing ExoS with active GAP, *Pa* ExoS-WT or *Pa* ExoS-ADPRT(−), and compared with alterations induced by *Pa* ExoS-GAP(−). MTC or MTLn3 cell morphology was examined after a 1 hr 45 min co-culture period, which was previously found to allow detection of low levels of ExoS translocation by IF, while limiting toxic effects of ExoS on cell morphology (Bridge et al., 2012). Following co-culture, cells were stained for extracellular or intracellular *Pa*, HA to detect ExoS effector translocation, and actin to detect alterations in cytoskeletal structure.

**Fig. 3.**
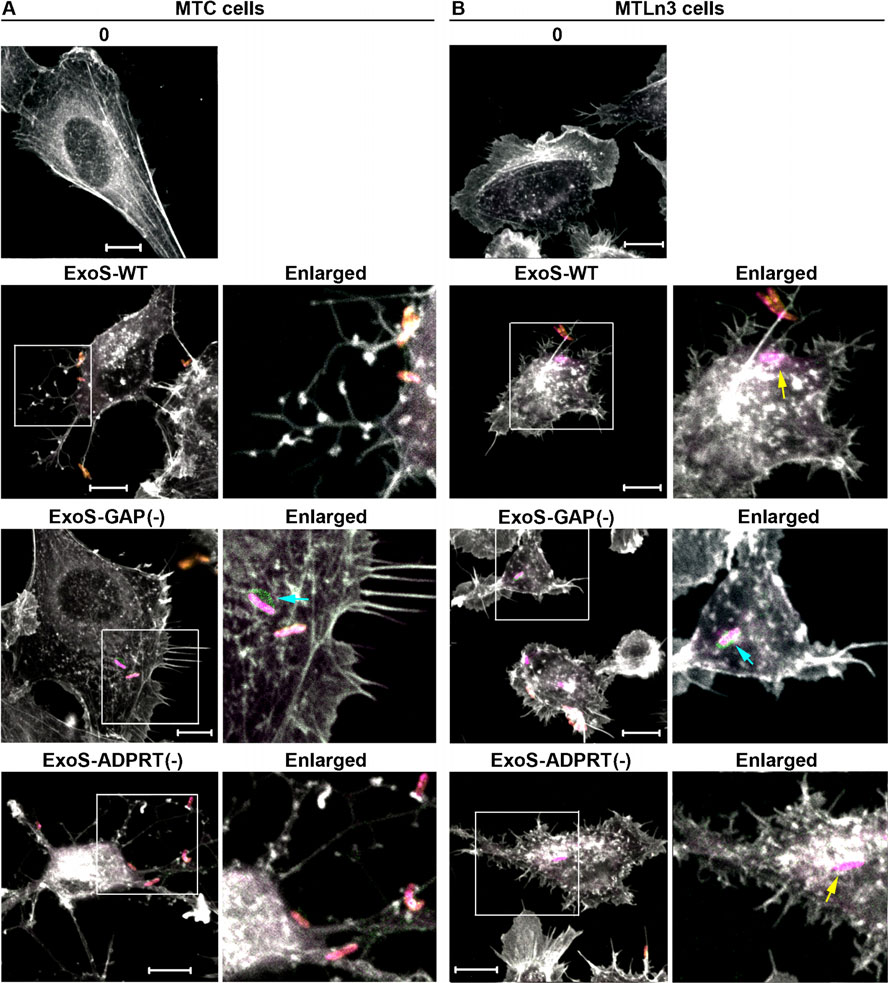
ExoS-GAP activity differentially alters MTC and MTLn3 cell morphology. (**A**) MTC or (**B**) MTLn3 cells were co-cultured for 1 hr 45 min with *Pa* expressing active GA, ExoS-WT or ExoS-ADPRT(−); inactive GAP, *Pa* ExoS-GAP(−); or with no *Pa* (0). Following co-culture cells were stained for extracellular *Pa* (yellow + pink), then fixed and permeabilized and stained for intracellular *Pa* (pink). ExoS effector was detected using an anti-HA antibody (green), and F-actin was stained using phalloidin (white). Scale bars: 10 µm. Regions of images enclosed in boxes were enlarged to allow better visualization of alterations in actin leading edge architecture following co-culture with *Pa* expressing the indicated ExoS effector. In enlarged images, intracellular *Pa* are indicated with yellow arrows, and intracellular *Pa* secreting ExoS effector are indicated with blue arrows. Results are representative of four independent experiments.

Distinctly different alterations in MTC and MTLn3 cell morphology were observed following co-culture with *Pa* expressing ExoS-WT (Fig. 3A,B, ExoS-WT panels). Translocation of ExoS-WT caused MTC cells to contract and leading edge architecture was replaced with branched actin filaments that remained adherent to cell matrix. Translocation of ExoS-WT into MTLn3 cells caused loss of actin demarcation of the lamellipodium, and instead actin-spiked projections protruded from within the lamellipodium. *Pa* expressing ExoS-WT was also internalized more efficiently into MTLn3 cells than MTC cells. Consistent with *Pa* ExoS-WT and *Pa* ExoS-ADPRT(−) both having GAP activity, morphological alterations caused by these two strains were similar. As with *Pa* ExoS-WT, *Pa* ExoS-ADPRT(−) caused MTC cells to contract, while branched actin filaments remained adherent cell matrix (Fig. 3A, ExoS-ADPRT(−) panels). In MTLn3 cells, *Pa* ExoS-ADPRT(−) caused actin-spiked projections to protrude from within the lamellipodium, similar to that observed in response to *Pa* ExoS-WT (Fig. 3B, ExoS-ADPRT(−) panels).

Inactivation of ExoS-GAP activity led to somewhat similar morphological outcomes in MTC and MTLn3 cells. Long, non-branching actin filaments projected from the lamellipodium of MTC cells in response to *Pa* ExoS-GAP(−), while the cells retained their same general morphological shape (Fig. 3A, ExoS-GAP(−) panels). Long actin filaments were also observed to project from the lamellipodium of MTLn3 cells in response to *Pa* ExoS-GAP(−), but the leading edge of MTLn3 cells often became distorted, as observed in Fig. 3B, ExoS-GAP(−) panels. Inactivation of ExoS-GAP increased *Pa* internalization into both MTC and MTLn3 cells, and this was accompanied by detection of ExoS-GAP(−) effector secretion by internalized *Pa* in both cell lines (Fig. 3A,B, enlarged ExoS-GAP(−) panels, marked by blue arrows). In summary, MTC and MTLn3 cells responded differently to *Pa* when ExoS-GAP was active but produced similar morphological outcomes when ExoS-GAP was inactive. The most severe morphological alterations caused by ExoS-GAP in both cell lines were alterations in actin organization affecting leading edge or lamellipodium architecture. These results are consistent with ExoS-GAP activity responding to differences in migratory properties of non-metastatic MTC and metastatic MTLn3 cells.

### Metastatic properties of MTLn3 cells enhance *Pa* internalization

**Fig. 4.**
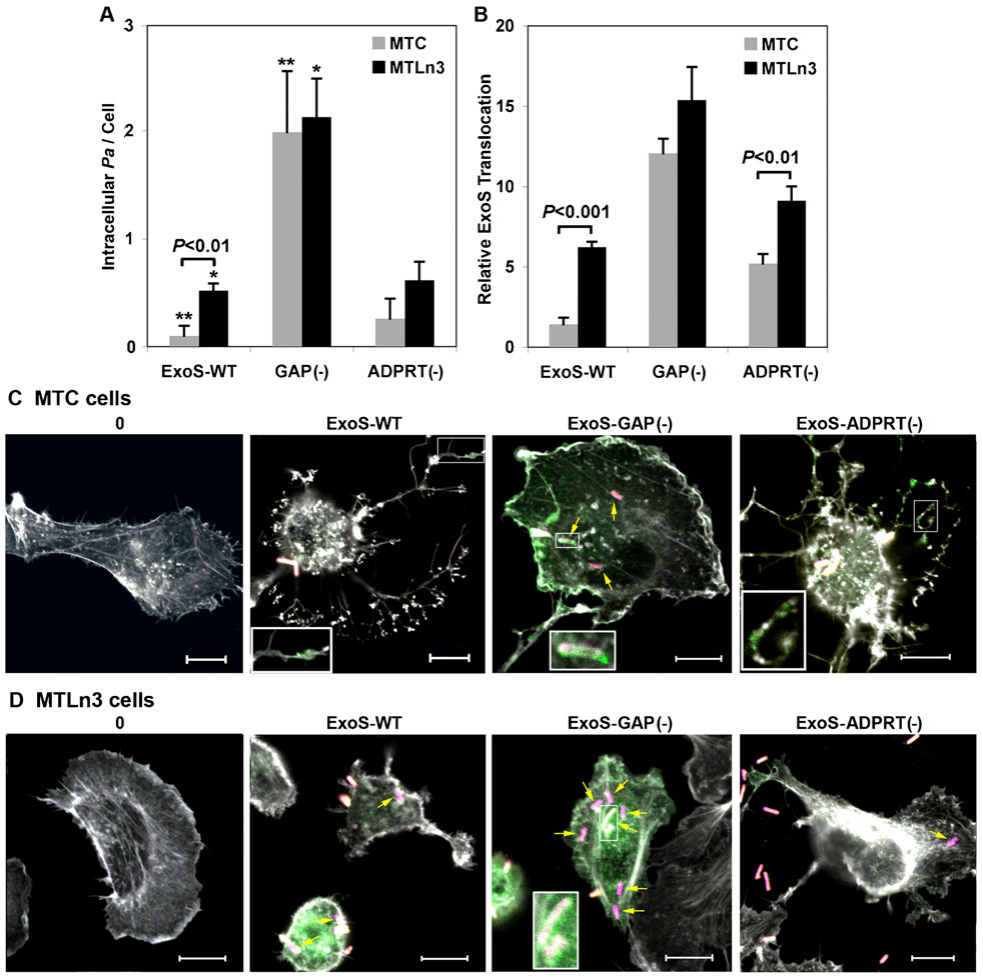
Metastatic properties of MTLn3 cells enhance *Pa* internalization. (**A**) MTC and MTLn3 cells were co-cultured for 1 hr 45 min to 3 hr in four independent experiments with *Pa* ExoS-WT, *Pa* ExoS-GAP(−) or *Pa* ExoS-ADPRT(−). Cells were stained for extracellular or intracellular *Pa* as in Fig. 3, and *Pa* internalization was visually enumerated based on *Pa* association with an average of 140 MTC or MTLn3 cells per strain. Results are expressed as the mean ± s.e.m. of *Pa* internalized per MTC or MTLn3 cell. Significant difference (*P*<0.01) in *Pa* ExoS-WT internalization in MTC and MTLn3 is indicated. Asterisks mark significant differences in *Pa* ExoS-WT and *Pa* ExoS-GAP(−) internalization in MTC cells (**P<0.001) or *Pa* ExoS-WT and *Pa* ExoS-GAP(−) internalization in MTLn3 cells (*P<0.01). (**B**) Compares ExoS data shown in Fig. 2B with *Pa* internalization data (A). Significant differences are indicated. (**C**) MTC and (**D**) MTLn3 cells were uninfected (0) or co-cultured for 3 hr with *Pa* ExoS-WT, *Pa* ExoS-GAP(−) or *Pa* ExoS-ADPRT(−). Cells were stained as in Fig. 3 for extracellular *Pa* (yellow + pink), intracellular *Pa* (pink, indicated with yellow arrows), ExoS effector (green), and actin (white). Insets show enlargement of regions where ExoS effector co-localizes with actin (MTC cells: ExoS-WT or ExoS-ADPRT(−) images), or where ExoS effector co-localizes with intracellular *Pa* (ExoS-GAP(−): MTC and MTLn3 cell images). Scale bars: 10 µm. Results are representative of four independent experiments, but images from a single experiment are compared in panels C and D.

The observation that *Pa* internalization was enhanced in association with increased ExoS translocation into MTLn3 cells led to our examination of the relationship between *Pa* internalization and ExoS translocation. *Pa* internalization into MTC or MTLn3 cells was visually enumerated, using IF staining and microscopy to identify intracellular *Pa*, in four independent experiments, where co-culture times ranged from 1 hr 45 min to 3 hr (Fig. 4A). Fig. 4B is included to allow direct comparisons of ExoS translocation based on immunoblot analysis with *Pa* internalization. *Pa* expressing ExoS-WT were internalized into MTLn3 cells 5-fold more efficiently than into MTC cells (*P*<0.01), and this corresponded with a 4.4-fold increase in ExoS-WT translocation into MTLn3 cells than MTC cells. When ExoS-GAP activity was inactivated, *Pa* internalization increased 20-fold (*P*<0.001) in MTC cells and 4-fold in MTLn3 cells (*P*<0.01), when compared to *Pa* ExoS-WT, essentially eliminating differences in *Pa* internalization between the two cell lines (Fig. 4A). No significant differences in *Pa* internalization were observed in either cell line between *Pa* ExoS-WT and *Pa* ExoS-ADPRT(−). Collectively, when *Pa* internalization and ExoS translocation were compared in MTC and MTLn3 cells following treatment with *Pa* ExoS-WT, *Pa* ExoS-GAP(−) or *Pa* ExoS-ADPRT(−) (comparisons of data represented in Fig. 4A,B) a positive (*r* = 0.96) correlation was observed, indicating that the efficiency of ExoS transfer into MTC and MTLn3 cells directly correlated with the efficiency of *Pa* internalization.

IF analysis of ExoS translocation into MTC and MTLn3 cells following a 3 hr co-culture found ExoS effector staining to be more intense in both cell lines (Fig. 4C,D), as compared to a 1 hr 45 min co-culture time in Fig. 3. Cells in general also became more rounded after the longer co-culture time. In MTC cells effector staining was observed to align with branched actin filaments that remained adherent to the cell matrix as cells contracted in response to *Pa* ExoS-WT or *Pa* ExoS-ADPRT(−) (Fig. 4C, insets show enlargement of ExoS-WT and ExoS-ADPRT(−) effector alignment with actin). Alignment of ExoS-WT or ExoS-ADPRT(−) effector with actin was not evident in MTLn3 cells, rather effector was diffusely distributed within the cell. Consistent with immunoblot analysis (Fig. 2A) effector staining reached highest intensity in both MTC and MTLn3 cells following co-culture with *Pa* ExoS-GAP(−) and was both diffuse within the cell and in close association with internalized *Pa* (Fig. 4C,D, ExoS-GAP(−) panels; intracellular *Pa* are marked by yellow arrows, insets show enlargement of close association of ExoS-GAP(−) effector with *Pa*). These studies provide further evidence that increased ExoS translocation into MTLn3 cells relates to *Pa* internalization and secretion of ExoS within cells, and that inactivation of ExoS-GAP equilibrates ExoS translocation in MTC and MTLn3 cells in conjunction with increased *Pa* internalization. The finding that ExoS-WT is secreted by internalized *Pa* within MTLn3 cells, whereas ExoS-WT is predominately T3S translocated into MTC cells by external *Pa*, also provides an explanation for the more efficient ADP-ribosylation of RalA in MTC cells, as ExoS would have an opportunity to gain access to membrane associated RalA during the T3S translocation process.

### Characterization of the target of ExoS-GAP activity that differentiates *Pa* infection in MTC and MTLn3 cells

ExoS-GAP activity targets Rho GTPases that regulate actin polymerization required for membrane protrusion during cell migration. MTLn3 cell motility has been well studied and differs from mesenchymal migration of most epithelial cells by the localization of RhoA, rather than Rac1, to protruding edges of migrating cells (El-Sibai et al., 2008). Rho and Rac activation are reciprocally balanced within cells during migration and determine whether tumor cells move in an amoeboid or mesenchymal manner (Sander et al., 1999; Sanz-Moreno et al., 2008). To test whether Rho activity defines the property that differentiates *Pa* infection in MTLn3 and MTC cells, ExoS translocation was monitored following treatment of both cell lines with Y-27632, an inhibitor of p160 Rho associated protein kinase (ROCK), which is a major downstream effector of Rho GTPase activity associated with tumor invasion (de Toledo et al., 2012). In this regard, previous studies in MTLn3 cells found that inhibition of either Rho GTPase or ROCK (Rho/ROCK) activity led to the reciprocal activation and localization of Rac1 to newly formed protrusions in MTLn3 cells (El-Sibai et al., 2008). As a control, both cell lines were treated with NSC23766, a Rac1 inhibitor, which is predicted to have no effect on T3S translocation in MTLn3 cells.

**Fig. 5.**
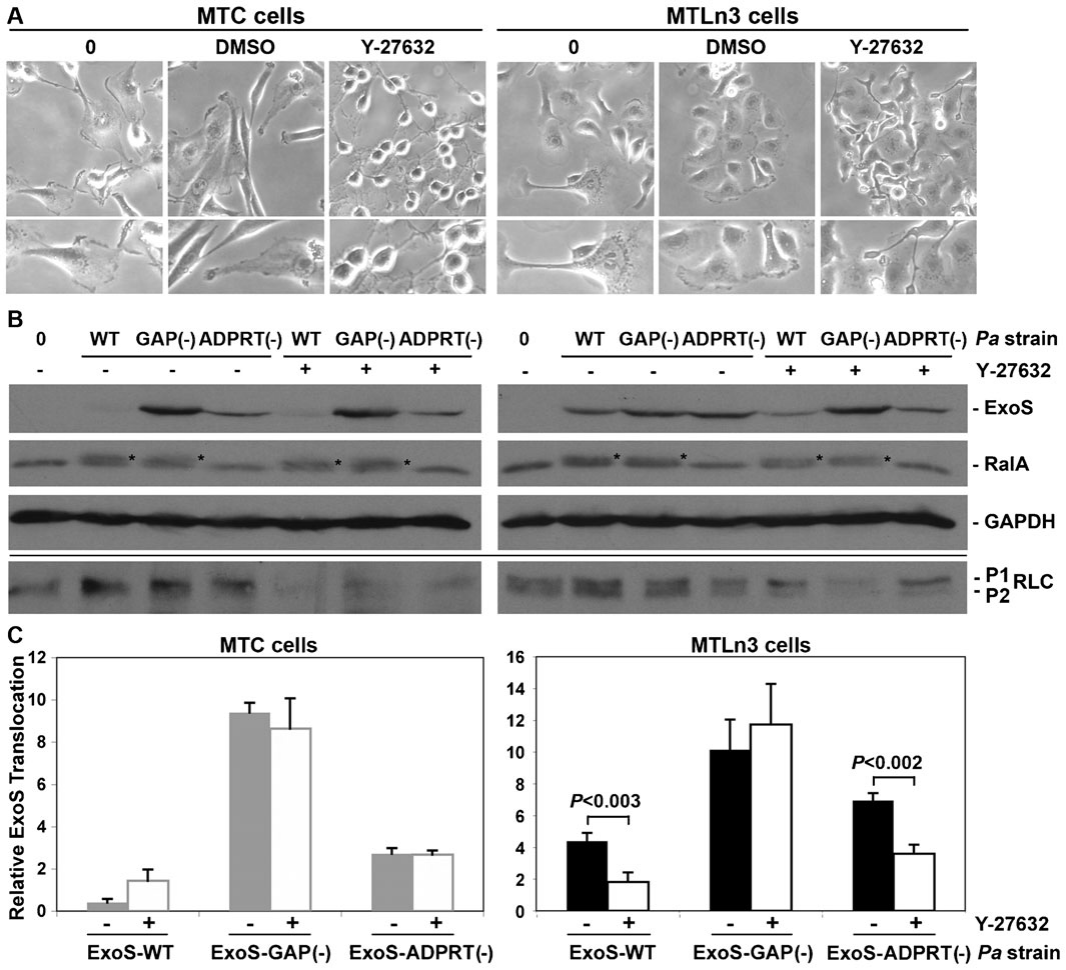
Inhibition of ROCK activity in MTLn3 cells decreased ExoS translocation. (**A**) MTC and MTLn3 cell morphology was examined by phase contrast microscopy following no treatment (0), serum starvation for 3 hr and treatment for 30 min with DMSO (control), or serum starvation and treatment for 30 min with 25 µM Y-27632. MTC cells severely contracted in response to ROCK inhibitor, while MTLn3 cells adapted an elongated morphology with a severely contracted trailing edge. Images were taken at 20× magnification. (**B**) Following treatment with ROCK inhibitor, MTC and MTLn3 cells were either uninfected (0) or co-cultured for 3.5 hr with *Pa* ExoS-WT, *Pa* ExoS-GAP(−) or *Pa* ExoS-ADPRT(−) under steady state conditions. Cells were washed, detached with trypsin, resuspended in Laemmli buffer, and ExoS translocation was examined by SDS-11%PAGE analysis based on equal protein loading. RalA modification served as a functional read-out of translocated ExoS-ADPRT activity, and is indicated by an asterisk. GAPDH served as a whole cell lysate protein loading control. Below line, samples were re-analyzed by SDS-14%PAGE based on equal protein and immunoblotted for mono- (P1) or di- (P2) phosphorylated myosin II regulatory light chain (RLC) to assess inhibition of ROCK activity by Y-27632. (**C**) T3S translocated ExoS was quantified by densitometry, and the mean ± s.e.m. of three independent experiments are represented. Significant differences in ExoS translocation following treatment of MTLn3 cells with ROCK inhibitor and co-culture with *Pa* ExoS-WT or *Pa* ExoS-ADPRT(−) are indicated.

In ROCK inhibition studies, MTC and MTLn3 cells were starved for 3 hr prior to the addition of 25 µM ROCK inhibitor, Y-27632, for 30 min. Phase contrast images show that treatment with ROCK inhibitor for 30 min caused severe contraction of MTC cell morphology. MTLn3 cells in comparison became more elongated in the presence of ROCK inhibitor, with the trailing edge becoming severely contracted. Following treatment with ROCK inhibitor, cells were either uninfected or co-cultured for 3.5 hr with *Pa* ExoS-WT, *Pa* ExoS-GAP(−), or *Pa* ExoS-ADPRT(−) in the presence of inhibitor under steady-state conditions. When ExoS translocation was assayed in MTC cells following treatment with ROCK inhibitor, no significant alteration in the ExoS translocation pattern was detected following co-culture with *Pa* ExoS strains as compared with non-ROCK inhibitor treated cells (Fig. 5B). In contrast, treatment of MTLn3 cells with ROCK inhibitor caused a significant decrease in translocation by *Pa* ExoS-WT (*P*<0.003) and *Pa* ExoS-ADPRT(−) (*P*<0.002), while causing no significant alteration in *Pa* ExoS-GAP(−) translocation. Myosin II regulatory light chain (RLC) is one of several substrates phosphorylated by ROCK, and when RLC phosphorylation was used to assess inhibition of ROCK activity by Y-27632, a decrease in both mono- (P1) and di-phosphorylated (P2) RLC were detected in cell lysates following treatment of MTC or MTLn3 cells with ROCK inhibitor and co-culture with *Pa* strains (Fig. 5B). Alternatively, when both cell lines were starved for three hours, and then treated with 50 µM NSC23766 for 12–16 hr to inhibit Rac1 activity prior to co-culture with *Pa* strains, no significant alteration in ExoS translocation in either MTC or MTLn3 cells was detected (data not shown). In summary, these experiments found that treatment of MTLn3 cells with ROCK inhibitor altered their ExoS translocation pattern to one similar to that of MTC cells, while treatment of MTC cells with ROCK inhibitor, or treatment of both cell lines with Rac1 inhibitor, caused no significant alterations in ExoS translocation. Collectively our results are consistent with activation of Rho/ROCK, but not Rac1, mediating increased translocation of ExoS-WT in highly metastatic MTLn3 cells, and highlight the ability of *Pa* ExoS-GAP activity to function as a biological probe to detect alterations in MTLn3 cell properties linked to metastasis.

## Discussion

The high susceptibility of cancer patients to *P. aeruginosa* infections is generally attributed to immunosuppression and systemic compromises associated with cancer treatments. As a result, the possibility that alterations at the tumor cell level might contribute to *Pa* infection remains relatively unexplored. In focusing on how metastatic properties of tumor cells might influence *Pa* infection, we found that ExoS translocation was more efficient in highly metastatic MTLn3 cells as compared to non-metastatic MTC cells. *Pa* is primarily an extracellular pathogen, but further analysis revealed that increased ExoS translocation into MTLn3 cells directly correlated with *Pa* internalization and secretion of ExoS within MTLn3 cells, which contrasts with conventional T3S translocation by extracellular *Pa* through a T3S membrane translocon channel. Differences in MTLn3 and MTC cell responsiveness to *Pa* ExoS related to reactivity to ExoS-GAP activity. In turn, inactivation of ExoS-GAP by an R146A mutation eliminated differences in the susceptibility of MTC and MTLn3 cell to *Pa* infection as assessed based on ExoS translocation and *Pa* internalization. ExoS-GAP activity targets Rho family proteins, and inhibition of ROCK activity, a downstream effector of Rho GTPase activity associated with tumor invasion (de Toledo et al., 2012), significantly decreased ExoS translocation into MTLn3 cells. The finding that Rho/ROCK signaling in MTLn3 cells enhanced *Pa* internalization, and that this resulted in increased ExoS transfer into MTLn3 cells identifies one property of metastatic tumor cells that can alter sensitivity to *Pa* infection.

The ability of *Pa*-ExoS to differentially infect MTC and MTLn3 cells based on their metastatic properties introduces the interesting possibility that reactivity to *Pa* ExoS might serve as a tool to predict or detect the metastatic potential of neoplastic tissue. Tumor progression to metastasis is a complex process that involves modulations of cell adhesion, migration, and extracellular matrix (Struckhoff et al., 2011). This complexity has complicated detection and delineation of the metastatic processes. The possibility that *Pa* ExoS can serve as a tool for assessing the metastatic potential of tumors is predicated on the ability of *Pa* to infect migrating (invasive) cells, and the ability of ExoS to target eukaryotic cell proteins that mediate tumor progression and metastasis, including Rho-GTPases, Ras family proteins, ERM proteins, vimentin and cyclophilin A (Struckhoff et al., 2011; Bos, 1988; Curto and McClatchey, 2004; Lee and Kim, 2010; Satelli and Li, 2011). Interestingly, the focus of this study became ExoS-GAP activity, which served as a functional probe to differentiate Rho GTPase activity in MTC and MTLn3 cells. Previous studies comparing Rac and Rho activity in MTC and MTLn3 cells found MTC cells to have decreased RhoA activity, increased Rac activity and to utilize Rac to produce membrane protrusions that mediate cell migration (El-Sibai et al., 2008). MTLn3 cells in comparison were characterized as having increased RhoA activity, decreased Rac activity, and to project Cdc42 mediated membranes protrusions during cell migration (El-Sibai et al., 2007; Yip et al., 2007). Notably, the phenotype of Rho/ROCK activation at the leading edge of MTLn3 cells could be switched to Rac1 driving cell protrusion by treatment with a ROCK inhibitor (El-Sibai et al., 2008), just as the phenotype of enhanced ExoS translocation in MTLn3 cells could be switched to low ExoS translocation by treatment with a ROCK inhibitor. The finding that *Pa* infection and ExoS translocation can differentiate Rac1 (non-metastatic) or RhoA (metastatic) properties of MTC or MTLn3 cells, and detect the reversal of these properties following treatment with ROCK inhibitor, draws attention parallels between *Pa* infection and tumor metastasis. Evidence that *Pa* ExoS-GAP activity can discriminate differences in Rho GTPases in tumor cells, beyond Rho or Rac activation, was provided by IF studies, which found MTC cells to contract but retain stably adhered branched actin filaments in response to *Pa* ExoS-GAP, which contrasted with the response of MTLn3 cells, where leading edge architecture was replaced with actin microspikes. The precise mechanisms underlying morphological differences in MTC and MTLn3 cell responsiveness to ExoS-GAP activity remain unknown, but they identify previously unrecognized differences in adhesion properties of MTC and MTLn3 cells, which might prove applicable to diagnosis of metastatic tumors.

The possibility that *Pa*-ExoS might also serve as a biological probe to dissect cellular mechanisms that underlie tumor metastasis is founded on interesting similarities between *Pa* infection and tumor development. For example, the finding that increased levels of ExoS transfer into metastatic MTLn3 cells related to *Pa* internalization draws attention to relationships between membrane properties that facilitate *Pa* internalization and those associated with tumor metastasis. *Pa* is predominately an extracellular pathogen, and susceptibility to infection and *Pa* internalization is influenced by epithelium integrity and polarity (Fleiszig et al., 1997; Plotkowski et al., 1999). In studies of MDCK epithelial cells, *Pa* internalization was enhanced in incompletely polarized monolayers in association with RhoA activation and decreased in highly polarized monolayers in conjunction with Cdc42 activation (Kazmierczak et al., 2004). In our studies, inhibition of ROCK activity significantly decreased ExoS translocation into MTLn3 cells, consistent with Rho/ROCK activation mediating enhanced *Pa* internalization in MTLn3 cells. Rho/ROCK signaling also mediates amoeboid migration and membrane blebbing that enhance tumor cell invasiveness (Struckhoff et al., 2011). The possibility that membrane blebbing might be a common basis for *Pa* internalization and tumor metastasis is supported by a recent study that identified the ability of *Pa* to enter, survive and replicate within membrane blebs of corneal epithelial cells, in an ExoS-ADPRT dependent manner (Angus et al., 2010). Membrane blebbing is a rapid, dynamic process, and a core element in bleb formation is retraction of the plasma membrane, which is mediated by signaling through Rho/ROCK and actinomycin contractility (Charras and Paluch, 2008). Thus, comparisons of tumor metastasis with *Pa* internalization find that both processes are facilitated by Rho/ROCK activation and by membrane bleb formation. The possibility that Rho/ROCK mediated membrane blebs might contribute to *Pa* internalization in MTLn3 cells is supported by the detection of blebbing in MTLn3 cells, but not MTC cells, following infection of with *Pa* ExoS-GAP(−) that maintains ExoS-ADPRT activity (supplementary material Fig. S1). Functional similarities between membrane blebs that mediate bacterial internalization and tumor metastasis have not previously been realized, and awareness of these similarities provides a new perspective on alterations in membrane properties that occur in association with tumor metastasis.

To summarize, in using metastatic MTLn3 cells and non-metastatic MTC cells as cell culture models to study *Pa* infection we were able to confirm that alterations at the tumor cell level can contribute to the establishment of *Pa* infection. Mechanistically enhanced *Pa* infection in MTLn3 cells related to Rho/ROCK activation, which drives cell migration and membrane plasticity during tumor cell invasion, and also facilitates *Pa* internalization. This is the first study to characterize a mechanism underlying the increased susceptibility of cancer patients to *Pa* infection at the cellular level. Reactivity to T3S translocated ExoS also allowed recognition of functional differences in Rho GTPase activity between MTC and MTLn3 cells that extend beyond Rac1 and Rho activation. Notably, commonalities observed between tumor development and *Pa* infection, along with the finding that reactivity to *Pa* ExoS can differentiate non-metastatic and metastatic properties of MTC and MTLn3 cells, introduces the notion that *Pa* ExoS might serve as a biological tool to assess the metastatic properties of neoplastic tissue. While further studies are required to determine how *Pa* ExoS will respond to different tumorigenic states, the ability of ExoS to target and alter the function of multiple tumor-associated proteins provides the diversity needed accommodate tumor heterogeneity.

## Materials and Methods

### Bacterial strains

*P. aeruginosa* strain PA103ΔUT is T3S effector-less derivative of strain PA103 that maintains an intact T3S system (Vallis et al., 1999). This strain was used to express: 1) plasmid encoded HA-tagged wild type ExoS, 2) ExoS with mutations that inactivate its GAP or ADPRT activities, or 3) a pUCP vector control, as previously described (Bridge et al., 2012). *Pa* strains were grown in ExoS induction medium (Iglewski et al., 1978) for 14–16 hr and were washed twice in cell culture medium prior to infection of eukaryotic cells.

### Eukaryotic cell culture

Rat mammary adenocarcinoma MTLn3 and MTC cell lines were obtained from John Condeelis (Albert Einstein College of Medicine, Bronx NY) and Jonathan Backer (Albert Einstein College of Medicine), respectively. Both cell lines were cultured in Minimal Essential Medium, Alpha (MEM; Cellgro, Manassas VA), containing nonessential amino acids (Cellgro), 10% fetal bovine serum (FBS; Hyclone, Logan UT), 100 U/ml penicillin, and 100 µg/ml streptomycin (Hyclone).

### Analysis of *Pa*-T3S translocation

*Pa*-T3S translocation was assayed by monitoring transfer of the T3S effector, ExoS, and T3S translocon protein, PopB, into membrane fractions following co-culture with *Pa* strains. For these analyses, MTC and MTLn3 cells were seeded at 2×10^4^ and 2.5×10^4^ cells/ml, respectively, in 60 mm dishes (Corning Inc., Corning, NY) and grown for 48 hr. Prior to addition of bacteria, cells were washed with Dulbecco's phosphate buffered saline (DPBS; Hyclone) and starved for 3–12 hr in MEM containing 0.35% bovine serum albumin (BSA; Sigma–Aldrich, St. Louis MO) and 12 mM HEPES (pH 7.4) (starvation medium). T3S translocation was analyzed following co-culture of MTC and MTLn3 cells for 0 to 3.5 hr with 10^7^ CFU/ml *Pa* in MEM containing 0.6% BSA. Following co-culture, bacteria were removed, cells were washed twice with DPBS containing 200 µg/ml ciprofloxacin, and then incubated with 0.25% trypsin–1 mM EDTA (Hyclone) to detach cells and degrade extracellular proteins. An aliquot of cells was removed and assayed for viability using trypan blue staining. Cells were washed with DPBS and fractionated as previously described (Rocha et al., 2005). An aliquot of the membrane fraction was removed and analyzed for total protein (BCA Protein Assay; Pierce, Rockville IL). Transfer of ExoS and PopB into membrane fractions was determined by SDS-PAGE and immunoblot analysis, based on equal protein loading. Protein bands were quantified by densitometry analysis of immunoblots using ImageJ 1.40 g software (http://rsbweb.nih.gov/ij), as previously described (Bridge et al., 2010; Fraylick et al., 2001). RalA served as a membrane fraction loading control, and ADP-ribosylation of RalA provided a functional assay of T3S translocated ExoS-ADPRT activity (Fraylick et al., 2002a). Antibodies used for immunoblot analysis included: anti-HA (Covance Research, Princeton NJ) to detect HA-tagged ExoS effectors, anti-PopB (provided by Joseph Barbieri, Medical College of Wisconsin, Milwaukee, WI), and anti-RalA (BD-Transduction, San Jose CA).

To visualize T3S effector translocation and *Pa* internalization by IF microscopy, MTC and MTLn3 cells were seeded at 2.4×10^4^ cells/ml in chamber slides (Nalge Nunc International, Rochester NY) for 24 and 48 hr, respectively. Cells were starved for 3 hr and co-cultured for 0 to 3 hr with 10^7^ CFU/ml *Pa* in MEM containing 5% FBS (steady state conditions) (El-Sibai et al., 2008; Shestakova et al., 1999). Following co-culture, cells were washed twice in DPBS and processed for IF analysis, as previously described (Bridge et al., 2012; Bridge et al., 2010). Briefly, external *Pa* were stained using anti-*Pa* LPS (obtained from Joseph Lam, University of Guelph, Guelph, Ontario) and visualized using a Qdot 655 goat anti-rabbit conjugate (Invitrogen). Cells were then fixed, permeabilized, blocked, and both intracellular and extracellular *Pa* were stained with anti-*Pa* LPS followed by an Alexa Fluor 647 conjugate (Invitrogen) (Bridge et al., 2012). Internal *Pa* were differentiated by the absence of Qdot 655 staining. Intracellular or extracellular bacterial localization was visually enumerated. HA-tagged ExoS effectors were detected using anti-HA and an anti-mouse Alexa Fluor 488 conjugate. F-actin was detected using Phalloidin-TRITC (Sigma–Aldrich). Images were examined using a Plan-Apochromat 63×/1.40 oil objective on a Zeiss Imager.Z1 LSM 510 confocal microscope (Jena, Germany) and exported to Adobe Photoshop CS4 Extended (Adobe Systems Inc., San Jose, CA) as 8 bit TIFF files. Channels in IF images were pseudocolored as indicated to improve visibility.

### Inhibition Rho and Rac activity

To assess the role of Rho and Rac in *Pa*-T3S translocation, MTC and MTLn3 cells were seeded in 60 mm dishes, as described above, and starved for 3 hr prior to addition of inhibitors. Rho activity was inhibited by treating cells for 30 min in starvation medium with 25 µM Y-27632 (Cayman Chemical Company, Ann Arbor, MI), an inhibitor of p160 Rho associated protein kinase (ROCK), which is a downstream effector of Rho GTPase activity known to influence cell invasion (de Toledo et al., 2012). Rac1 activity was inhibited by treating cells for 12–16 hr with 50 µM NSC23766 (EMD Chemicals, Gibbstown, NJ) in starvation medium. Effects of the drugs on cell morphology were visualized with a LD A-Plan 20×/0.3 Ph1 objective on a Zeiss Axiovert 200 microscope (Jena, Germany). Images were exported to Adobe Photoshop CS4 Extended (Adobe Systems Inc.) as TIFF files and cropped.

Following treatment with inhibitor, cells were co-cultured with *Pa* strains for 3.5 hr in the presence of inhibitor in MEM containing 5% FBS. Cells were then washed twice with DPBS containing 200 µg/ml ciprofloxacin and detached with trypsin. An aliquot of cells was lysed and analyzed for total protein (BCA Protein Assay, Pierce). Remaining cells were resuspended in DPBS containing ciprofloxacin and lysed in Laemmli sample buffer. Whole cell lysates were analysed by SDS-11% PAGE and immunoblot analysis as above, using anti-HA to detect T3S translocated ExoS effectors, anti-RalA to assess T3S translocated ExoS-ADPRT activity, and anti-glyceraldehyde 3-phosphate dehydrogenase (GAPDH) (Millipore, Temecula CA) to monitor protein loading of whole cell lysates. To detect inhibition of ROCK activity by Y-27632 in conjunction with alterations in ExoS translocation, whole cell lysates were re-analyzed by SDS-14% PAGE, and myosin II light chain phosphorylation was detected using an affinity purified rabbit anti-phospho Thr18/Ser19 RLC antibody (Chew et al., 1998). ExoS effector translocation was quantified by densitometry using ImageJ 1.40 g software.

### Statistical analysis

JMP Version 9 Software (SAS Institute, Cary, NC) was used for statistical analysis, and significance was determined by a two-sided alpha level set at *P*<0.05. One-way ANOVAs were used to evaluate differences among *Pa* strains between cell lines. Multiple comparisons were analyzed with a Tukey's HSD test for ExoS translocation and *Pa* internalization. A T-test was used to evaluate differences in ROCK and Rac1 inhibitor studies.

## Supplementary Material

Supplementary Material
