# Supplementary material for: Metastatic MTLn3 and non-metastatic MTC adenocarcinoma cells can be differentiated by Pseudomonas aeruginosa
Source: Biol Open. 2013 Jul 3;2(9):891–900. doi: 10.1242/bio.20133632 (PMC3773335; doi:10.1242/bio.20133632)
Supplement: Supplementary Material [file supp_2_9_891__index.html]

Metastatic MTLn3 and non-metastatic MTC adenocarcinoma cells can be differentiated by Pseudomonas aeruginosa — Metastatic MTLn3 and non-metastatic MTC adenocarcinoma cells can be differentiated by Pseudomonas aeruginosa — Supplementary Material 

# Metastatic MTLn3 and non-metastatic MTC adenocarcinoma cells can be differentiated by *Pseudomonas aeruginosa*

## 

**Files in this Data Supplement:**

- Supplementary Material - Matthew J. Novotny et al. doi: 10.1242/bio.20133632
