# Supplementary material for: Metastatic MTLn3 and non-metastatic MTC adenocarcinoma cells can be differentiated by Pseudomonas aeruginosa
Source: Biol Open. 2013 Jul 3;2(9):891–900. doi: 10.1242/bio.20133632 (PMC3773335; doi:10.1242/bio.20133632)
Supplement: Supplementary Material [file supp_bio.20133632_bio.20133632-s1.pdf]

Biology Open

Supplementary Material

Matthew J. Novotny et al. doi: 10.1242/bio.20133632

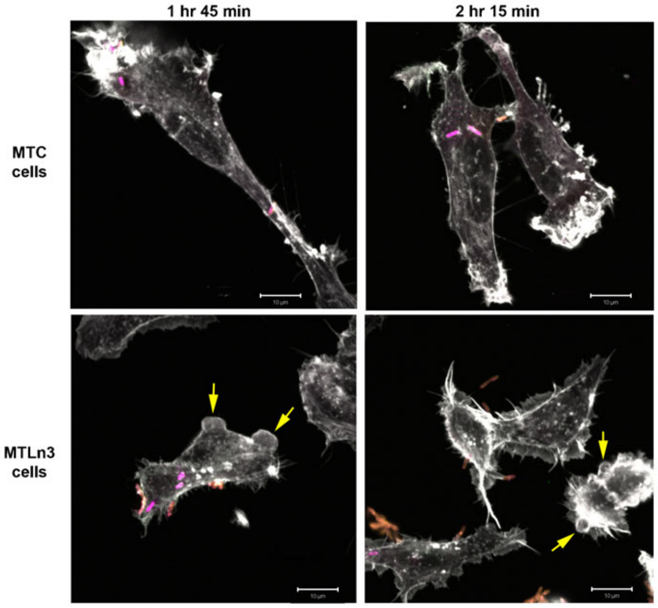

Fig. S1. Detection of blebbing in MTLn3 cells following infection *Pa* ExoS-GAP(−). MTC and MTLn3 cells were co-cultured for the indicated times with *Pa* expressing ExoS with a R146A mutation that inactivates ExoS GAP activity but does not alter ExoS ADPRT activity. Cells were fixed and stained for extracellular *Pa* (yellow + pink) or intracellular *Pa* (pink) and actin (white). Yellow arrows mark blebbing that occurs in MTLn3 but not MTC cells following exposure to ADPRT active ExoS. Scale bars: 10 µm.
